# Effects of Mindfulness-Based Stress Reduction on employees’ mental health: A systematic review

**DOI:** 10.1371/journal.pone.0191332

**Published:** 2018-01-24

**Authors:** Math Janssen, Yvonne Heerkens, Wietske Kuijer, Beatrice van der Heijden, Josephine Engels

**Affiliations:** 1 Occupation & Health Research Group, HAN University of Applied Sciences, Nijmegen, the Netherlands; 2 Institute for Management Research, Radboud University, Nijmegen, the Netherlands; 3 Open University of the Netherlands, Heerlen, the Netherlands; 4 Kingston University, London, United Kingdom; University of Oxford, UNITED KINGDOM

## Abstract

**Objectives:**

The purpose of this exploratory study was to obtain greater insight into the effects of Mindfulness-Based Stress Reduction (MBSR) and Mindfulness-Based Cognitive Therapy (MBCT) on the mental health of employees.

**Methods:**

Using PsycINFO, PubMed, and CINAHL, we performed a systematic review in October 2015 of studies investigating the effects of MBSR and MBCT on various aspects of employees’ mental health. Studies with a pre-post design (i.e. without a control group) were excluded.

**Results:**

24 articles were identified, describing 23 studies: 22 on the effects of MBSR and 1 on the effects of MBSR in combination with some aspects of MBCT. Since no study focused exclusively on MBCT, its effects are not described in this systematic review. Of the 23 studies, 2 were of high methodological quality, 15 were of medium quality and 6 were of low quality. A meta-analysis was not performed due to the emergent and relatively uncharted nature of the topic of investigation, the exploratory character of this study, and the diversity of outcomes in the studies reviewed. Based on our analysis, the strongest outcomes were reduced levels of emotional exhaustion (a dimension of burnout), stress, psychological distress, depression, anxiety, and occupational stress. Improvements were found in terms of mindfulness, personal accomplishment (a dimension of burnout), (occupational) self-compassion, quality of sleep, and relaxation.

**Conclusion:**

The results of this systematic review suggest that MBSR may help to improve psychological functioning in employees.

## Introduction

Given their potential benefits for physical and mental health as well as social relations [1, 2], interest is increasing internationally in mindfulness interventions in the workplace [3]. This is also true for the Netherlands, where many work environments are characterized by high productivity targets, overtime, high work pressure, customer aggression, temporary employment contracts, continuous organizational changes, job uncertainty, employee shortages, and little autonomy [4].

Mindfulness is related to meditation, but the terms are not synonymous. Moreover Mindfulness consists of formal meditation exercises (e.g., paying attention to the body, lying on the ground, or walking slowly with a sense of awareness of one’s surroundings) as well as informal exercises (e.g., paying full attention to what one is doing or experiencing at a certain moment) [5].

Mindfulness interventions vary in delivery mode (face-to-face, online) and target population (clinical populations with major depression, anxiety disorders, borderline personality disorders, chronic pain, or eating disorders [6] and non-clinical populations such as students and employees seeking to enhance their subjective well-being). They can range in duration from long term (e.g., eight weekly 2.5-hour sessions, eight-hour daylong retreats, one 2.5-hour session per month for 10 months [7]) to short term (e.g., four weekly 30-minute sessions [8]). The intensity of interventions can vary too, from high dose (e.g., eight weekly 3-hour sessions and 45 minutes of daily mindfulness practice [9]) to low dose (e.g., 30-minute sessions and 15–20 minutes of daily mindfulness practice [8]).

Mindfulness interventions in the workplace target workplace functioning: reducing stress and improving decision-making, productivity, resilience, interpersonal communication, organizational relationships, perspective-taking, and self-care [10]. This great diversity in mindfulness interventions makes it difficult to compare the efficacy of such interventions.

There are many hypotheses about the mechanisms underlying mindfulness practice that lead to different mental health outcomes. One hypothesis is that exposure to or willingness to experience difficult emotions (e.g., anxiety, distress, anger), awareness of these emotions, and observation of these emotions allow people to dis-identify with and better regulate difficult emotions [5, 11]. Another is that awareness of thoughts, awareness of bodily sensations, and self-compassion help people to deal with stress [5].

This systematic review will evaluate the effects of two types of group-based mindfulness interventions—Mindfulness-Based Stress Reduction (MBSR) and Mindfulness-Based Cognitive Therapy (MBCT)–on employees’ mental health.

### Definition of mindfulness

The concept of mindfulness has existed in Buddhist traditions for 2,500 years. Mindfulness meditation is an attitude and a method for reducing personal suffering and developing insight, compassion, and wisdom [12]. In contemporary psychology, mindfulness is seen as a means of increasing awareness and responding optimally to mental processes that contribute to emotional distress and maladaptive behavior [6].

Many definitions of mindfulness have been posited in the psychological literature [6, 13–15]. Marlatt and Kristeller [13] described mindfulness as “bringing one’s complete attention to the present experience on a moment-to-moment basis.” According to Brown and Ryan [14], “mindfulness can be considered an enhanced attention to and awareness of current experience or present reality.” ‘Awareness’ refers to the individual’s consciousness of what they are experiencing, without those experiences being the center of attention. ‘Attention’ is the process of focusing conscious awareness on specific experiences.

Bishop et al. [6] proposed a working definition of mindfulness with two components. The first concerns the self-regulation of attention, which is focused on the immediate experience in the present. The second involves having an open, curious, and accepting attitude towards that experience. Kabat-Zinn [15] described mindfulness as “the awareness that emerges through paying attention, on purpose, in the present moment, and non-judgmentally to the unfolding of experience moment by moment” (p. 145). This awareness can be aimed at internal experiences (bodily sensations, feelings/emotions, and thoughts) and external experiences (what one sees, hears, smells, tastes, and touches).

Shapiro and Carlson [16] defined mindfulness as “the awareness that arises through intentionally attending in an open, caring, and discerning way.” This definition contains three interrelated elements. The first element—intention—involves reflecting on one’s personal goals and values, and paying attention to the most important things in accomplishing and upholding them. The second element, attention (i.e., attending to experiences in the here and now) is a prerequisite for seeing clearly. Finally, while intention refers to *why* we are paying attention, the third element—attitude—relates to *how* we pay attention. It refers not to an attempt to change things, but to an effort to relate to them in a non-judgmental way, with curiosity and compassion [10].

In this review article, we adopt Shapiro and Carlson’s [16] definition, which is an elaboration of the commonly accepted definition by Kabat-Zinn [15].

### Interventions based on Kabat-Zinn’s mindfulness training

The most common form of secular mindfulness-based training is Mindfulness-Based Stress Reduction (MBSR) training [17], developed by Kabat-Zinn [18]. MBSR aims to alleviate suffering [19] and was originally developed for patients with chronic pain. It consists of eight 2.5-hour weekly sessions and one 7-hour day of silence. A very important part of the training is the homework: 45 minutes of daily exercise at home, 6 days a week, with the support of CDs and set tasks. MBSR includes:

- the body scan (paying attention to what the body is feeling);
- sitting meditation (paying attention to breathing, sounds, thoughts, bodily sensations, feelings/emotions);
- simple movement exercises such as walking or standing meditation, or lying yoga exercises (paying attention to what the body is feeling; exploring and accepting borders);
- informal meditation exercises: paying full attention to daily activities (e.g., brushing one’s teeth, taking a shower, eating).

Mindfulness-Based Cognitive Therapy (MBCT), which was developed by the cognitive behavior therapists Segal, Williams, and Teasdale [20] on the basis of MBSR, is often used to prevent relapse into depression [21–23]. In workplace settings, the focus is on distressing emotional states rather than on clinical depression (e.g., see the pre-post study by Ruths et al. [24]). MBCT has become the most important adaptation of MBSR [25], to which it is closely allied, although there are a few differences. The CT component of the program includes psycho-education about the nature of thoughts as mental events rather than facts, which fosters a decentered attitude towards one’s own thoughts: thoughts are not facts; I am not my thoughts. The link between thought and mood is made explicit in MBCT. Moreover, MBCT also introduces a ‘mini-meditation’, known as the three-minute breathing space.

Other interventions incorporate mindfulness training as well, including Dialectical Behavior Therapy for the treatment of clients with a borderline syndrome [26], Acceptance and Commitment Therapy [27], and Mindfulness-Based Relapse Prevention [28]. As mindfulness is only one component of the treatment in these interventions, they are not included in this review.

MBSR and MBCT interventions are typically modified to suit the context in which they are delivered (Table 1). Sessions can vary in number and duration; they may be face-to-face or online, and involve less or more homework; and the retreat day may be dropped. In this study, we include MBSR and MBCT interventions conducted in groups with at least four face-to-face 30-minute sessions.

**Table 1:** Summary of studies that examined the impact of Mindfulness-Based Stress Reduction (MBSR).

Study:	Characteristics:	Research:	Assessment instruments used	Outcomes
**1) Author(s)**	**1) N / amount of participants**	**1) Research design**		
**2) Quality**	**2) Type of non-clinical participant**	**2) Treatment group (TG) after drop out / Control group(s) (CG) after drop out / Drop-outs**		
	**3) Mean age**	**3) Moments of measurement**		
	**4) % male**	**4) Treatment given**		
1) Duchemin et al. [76]	1) N = 32	1) Randomized controlled study	1) Five Facets Mindfulness Questionnaire (FFMQ). Five subscales: observing; describing; acting with awareness; non-judging; non-reactivity (1)^1^	Significant increase in mindfulness: not reported. (1)
2) High Quality	2) Personnel from a surgical intensive care unit (SICU) of a large academic medical center: 69% are nurses.	2) TG: n = 16 / CG: n = 16, waiting-list control / Drop-outs: n = 0	2) Perceived Stress Scale (PSS) (3)	No significant changes. (3)
	3) Mean age TG and CG: 44.2	3) Moments of measurement: 1. at baseline (T1); 2. post class (T2)	3) Depression Anxiety Stress Scale (DASS-21), stress subscale (3)	DASS stress scores decreased 25% in the TG (significant) compared to a non-significant 13% decline in the CG. (3)
	4) % male: 12.5%	4) Shortened MBSR program (mindfulness combined with yoga and music): 8 weekly 1 h sessions, except week 5 session 2 hours; daily 20 minutes exercise	4) Maslach Burnout Inventory (MBI). Three subscales: emotional exhaustion; personal accomplishment; depersonalization (2)	No significant changes between pre- and post-intervention. (2)
			5) Professional Quality of Life (ProQOF) (11)	No significant changes between pre- and post-intervention. (11)
1) Huang et al. [87]	1) N = 144	1) Randomized controlled study	1) Chinese Health Questionnaire (CHQ-12): adapted from the General Health Questionnaire (GHQ-12): measure of psychological well-being (4)	Significant decrease in psychological distress in the TG compared to the CG on T2 and T3. The positive effects in the TG remained at T4 and T5. (4)^1^
2) High Quality	2) Employees of two large-scale manufacturing factories (inclusion criterion: poor mental health, defined by exhibiting three criteria: in the top tertile of the distribution in the CHQ for psychological distress; in the bottom tertile for the subscale of job control and in the top tertile for the subscale of job demands in the JCQ for job strain)	2) TG: n = 58 / CG: n = 54, waiting-list control / Drop-outs: n = 32	2) Checklist Individual Strength (CIS)	Significant reduction in prolonged fatigue in the TG compared to the CG on T3. The positive effects in the TG remained at T4 and T5.
	3) Mean age TG: 42.4 / CG: 42.7	3) Moments of measurement: 1. at baseline (T1); 2. at mid-intervention (T2); 3. post class (T3); 4. 4 weeks post-intervention (T4); 5. 8 weeks post-intervention (T5)	3) Perceived Stress Scale (PSS-10) (3)	Significant reduction in perceived stress in the TG compared to the CG on T3. The positive effects in the TG remained at T4 and T5. (3)
	4) % male TG: 50.0%; CG: 68.1%	4) MBSR program: 8 weekly 2 h sessions; daily 45 minutes home practice, 7 days per week; no daylong retreat	4) Job Content Questionnaire (JCQ): only the subscales of job control and job demands	Significant differences in job strain (increase in job control and decrease in job demands) between the TG and CG on T3. Only job demands showed a significant difference between TG and CG at T5.
1) Amutio et al. [7]	1) N = 42	1) Randomized controlled study	1) Five Facets Mindfulness Questionnaire (FFMQ) (1)	1) Significant increase in mindfulness in the TG compared to the CG on T2: mindfulness total; observing; describing; non-judging; non-reactivity. Significant improvement in the TG between T2 and T3. (1)
2) Medium Quality	2) Physicians, actively employed in public (42.9%) or private (52.4%) practice. 66.7% of the sample had a work experience of at least 10 years.	2) TG: n = 21 / CG: n = 21, waiting-list control / Drop-outs: n = 2	2) Smith Relaxation States Inventory (SRSI-3). Four subscales: basic relaxation; positive energy; mindfulness; transcendence (8)	2) Significant increase in all the relaxation dimensions in the TG compared to the CG on T2. Relaxation levels increased around 30% between T1 and T3. (8)
	3) Mean age TG and CG: 47.3	3) Moments of measurement: 1. at baseline (T1); 2. post class (T2) (TG and CG measures); 3. 10 months post-intervention (T3): no CG measures!		
	4) % male: 42.9%	4) Extended MBSR program: First phase: 8 weekly 2.5 h sessions; 8 h daylong retreat (28 h); Second phase: next ten months, one session of 2.5 h per month (25 h)		
1) Taylor et al. [89]	1) N = 59	1) Randomized controlled study	1) Program Evaluation Survey (formal presentations; group mindfulness practices; group discussions)	‘Moderate’ to ‘quite a lot’ of benefit regarding regulating emotions and understanding / practicing forgiveness, kindness and compassion
2) Medium Quality	2) Elementary (39) and secondary school (21) teachers. Years of teaching experience ranged from 3 to 35 years (M = 15.2)	2) TG: n = 26 / CG: n = 30, waiting-list control / Drop-outs: n = 3	2) Occupational stress (9 items); an additional single item to assess state-changes in teachers’ occupational stress over the course of the program (10)	Significant reduction in occupational stress in the TG compared to the CG at T2. The effect size at T2 was large (Cohen’s d = 0.90); a medium effect size at T3 (Cohen’s d = 0.61). Teachers in the TG compared to the CG reported greater stress reduction at T2. (10)
	3) Mean age TG and CG: 47	3) Moments of measurement: 1. at baseline (T1); 2. post class (T2); 3. 4-month post-intervention (T3)	3) Efficacy for regulating emotion at work (set of 9 items to assess teachers’ perceived self-efficacy) (13)	Efficacy beliefs changed from pre/post for teachers in the TG. Efficacy beliefs partially mediated reduction in occupational stress from T1 to T3. (13)
	4) % male: 10.0%	4) Mindfulness training program: 9 weeks, 11 separate sessions for 36 total contact hours; 16 h home practice	4) Santa Clara Brief Compassion Scale (4 items)	No significant results.
			5) Tendency to Forgive scale (TFF; 4 items; teachers’ general tendency to forgive others)	Tendency to forgive changed from pre/post for teachers in the TG. Tendency to forgive partially mediated reduction in occupational stress from T1 to T3.
			6) Situation-Specific Forgiveness	No significant results.
			7) Efficacy for Forgiving Others at Work (2 items)	No significant results.
			8) Qualitative data ‘Teachers’ Coping at Work’	A trend that teachers in the TG reported more adaptive strategies for coping with occupational stress.
			9) Qualitative data ‘Teacher Compassion for Challenging Student’	A trend that teachers in the TG evaluate challenging students in a more positive affective light.
1) Roeser et al. [88]	1) N = 113	1) Randomized controlled study (two combined studies)	1) Five Facets Mindfulness Questionnaire (FFMQ) (1)	Significant increase in mindfulness in the TG compared to the CG (at T2 and T3). (1)
2) Medium Quality	2) Elementary and secondary school teachers. Years of teaching experience ranged from 1 to 35 years (M = 14.9)	2) TG: n = 54 / CG: n = 59, waiting-list control / Drop-outs: n = 0	2) Focused attention and Working Memory Capacity (automatic version of the Operation Span Task)	Significant increase in focused attention and working memory capacity in the TG compared to the CG at T2 and T3.
	3) Mean age TG and CG: 46.9	3) Moments of measurement: 1. at baseline (T1); 2. post class (T2); 3. 3-month post-intervention (T3)	3) Occupational self-compassion. Modification of Neff’s global self-compassion items to teachers: including self-kindness, self-judgment, common humanity, isolation and over-identification; excluding mindfulness items (6)	Significant increase in occupational self-compassion in the TG compared to the CG (at T2 and T3). (6)
	4) % male: 11.0%	4) Mindfulness training program: 8 weeks, 11 separate sessions for 36 total contact hours; home practice	4) Occupational stress (9 items) (10)	Significant decrease in occupational stress in the TG compared to the CG (at T2 and T3). (10)
			5) Maslach Burnout Inventory (MBI) (2)	Significant decrease in burnout in the TG compared to the CG at T2 and T3. (2)
			6) State-Trait Anxiety Inventory (STAI), State subscale (9)	Significant decrease in anxiety in the TG compared to the CG at T2 and T3. (9)
			7) Beck Depression Inventory (BDI) (5)	Significant decrease in depression in the TG compared to the CG at T2 and T3. (5)
			8) Program evaluation	87% found it beneficial.
1) Wolever et al. [82]	1) N = 239	1) Randomized controlled study	1) Perceived Stress Scale (PSS) (3)	Significant reductions in perceived stress in the TG mindfulness (= TG1 + TG2) compared to the CG. Significant reductions in perceived stress in the TG3 compared to the CG. No significant differences between the TG mindfulness and the TG3 in perceived stress. (3)
2) Medium Quality	2) Employees of a national insurance carrier (inclusion criterion: 16 or higher on the 10-item Perceived Stress Scale)	2) TG1 (In-person mindfulness; conventional classroom): n = 32 / TG2 (Online mindfulness; virtual classroom): n = 50 / TG3 (Yoga): n = 76 / CG: n = 47 / Drop-outs: 34 (TG1: n = 12; TG2: n = 2; TG3: n = 14; CG: n = 6)	2) Pittsburgh Sleep Quality Index (PSQI): general sleep quality; sleep latency; sleep duration; habitual sleep efficiency; sleep disturbances; the use of medication to sleep; daytime sleep-related dysfunction over the past month (7)	Significant reductions in sleep difficulty in the TG mindfulness (= TG1 + TG2) compared to the CG. Significant reductions in sleep difficulty in the TG3 compared to the CG. No significant differences between the TG mindfulness and the TG3 in sleep difficulty. (7)
	3) Mean age TG1, TG2, TG3 and CG: 42.9; TG1: unknown; TG2: unknown; TG3: unknown; CG: unknown	3) Moments of measurement: 1. at baseline; 2. post class	3) Center for Epidemiological Studies Depression Scale (CES-D) (5)	No significant results in depression were found. The TG3 reported significant less current pain than the CG. (5)
	4) % male: 23.4%	4) Shortened MBSR program, Mindfulness at work: 12 weekly 1 h sessions; 2 hours mindfulness practice intensive at week 10	4) Work Limitations Questionnaire (WLQ): a measure of health-related decrements in ability to perform job roles	No significant results were found.
			5) Cognitive and Affective Mindfulness Scale-Revised (CAMS-R) (1)	Significant increases in mindfulness in the TG mindfulness (= TG1 + TG2) compared to the CG. No significant differences between the TG3 and the CG, or between the TG mindfulness and the TG3. (1)
1) Pipe et al. [81]	1) N = 33	1) Randomized controlled study	1) Symptom Checklist 90-Revised (SCL-90-R) Three global distress indices: Global Severity Index (GSI: assessing overall psychological distress); Positive Symptom Distress Index (PSDI: assessing symptom intensity); Positive Symptom Total (4)	A significant reduction between T1 and T2 in psychological distress (stress, anxiety, mood) in the TG. No significant decrease in the CG. Significant improvement in the PSDI (assessing symptom intensity) and GSI (assessing overall psychological distress) in the TG compared to the CG. (4)
2) Medium Quality	2) Nursing leaders from a healthcare system	2) TG: n = 15 / CG: n = 17, waiting-list control / Drop-outs: n = 1	2) Caring Efficacy Scale: measure about one’s ability to express a caring orientation and establish a caring environment with patients	No significant difference in change from baseline caring efficacy between the TG and the CG.
	3) Mean age TG: 50,2; CG: 49,4	3) Moments of measurement: 1. at baseline (T1); 2. weeks post-intervention (T2); 3. 1 year post-intervention (eliminated, making it possible for CG to follow the training) (T3)		
	4) % male: 3,3%	4) Shortened MBSR program: 5 weekly 2 h sessions; daily 30 minutes exercise		
1) Klatt et al. [78]	1) N = 45	1) Randomized controlled study	1) Mindfulness Attention Awareness scale (MAAS) (1)	Significant increase in mindfulness in the TG compared to the CG. (1)
2) Medium Quality	2) Healthy working adults at a university: research assistants (31%), midlevel management (29%) and faculty employed (13%)	2) TG: n = 22 / CG: n = 20, waiting-list control / Drop-outs: n = 3	2) Perceived Stress Scale (3)	Significant reductions in perceived stress in the TG compared to the CG. (3)
	3) Mean age TG: 43.41; CG: 46.50	3) Moments of measurement: 1. at baseline; 2. post class	3) Pittsburgh Sleep Quality Index (PSQI) (7)	Subjective sleep quality, sleep latency, sleep disturbances and daytime dysfunction significantly improved in the TG; positive changes in subjective sleep quality, sleep disturbances and daytime dysfunction, although not significant (respectively: *p* = 0.07; *p* = 0.06; *p* = 0.07), for the CG; significant changes in the global sleep scores for the TG and for the CG. (7)
	4) % male: ± 25%	4) Shortened MBSR program: 6 weekly 1 h sessions; daily 20 minutes exercise, 6 days per week		
1) Cohen-Katz et al. Part II [73]: **the same study as Cohen-Katz et al. Part III [74]**	1) N = 27	1) Randomized controlled study	1) Mindfulness Attention Awareness Scale (MAAS) (1)	Significant increase in mindfulness in the TG compared to the CG between T1 and T2. Significant changes in the TG between T1 and T2, and between T1 and T3. (1)
2) Medium Quality	2) Nurses (90%); persons employed in pastoral care, respiratory therapy and social work	2) TG: n = 12 (T2) / CG: n = 13 (T2); waiting-list control / Drop-outs: n = 2	2) Maslach Burnout Inventory (MBI) (2)	Significant reduction of emotional exhaustion in the TG compared to the CG between T1 and T2. Also a significant reduction of emotional exhaustion in the TG between T1 and T2, and between T1 and T3. Lower depersonalization for the TG compared to the CG between T1 and T2, almost significantly (*p* = 0.06). Significant higher levels of personal accomplishment in the TG than in the CG between T1 and T2. (2)
	3) Mean age TG and CG: 46; TG: unknown; CG: unknown	3) Moments of measurement: 1. at baseline (T1); 2. post class (T2); 3. 3-month post-intervention (T3)	3) Brief Symptom Inventory (BSI) (psychological distress) (4)	No significant results were found. (4)
	4) % male: 0%	4) MBSR program: 8 weekly 2.5 h sessions; daily home practice, 6 days per week; daylong retreat		
1) Cohen-Katz et al. Part III [74]: **the same study as Cohen-Katz et al. Part II [73]**	1) N = 27	1) Randomized controlled study	Qualitative data: Getting to know you (15 participants): *questions about challenges / stressors; relaxation; medical*, *behavioral*, *emotional or mental problems; abuse*	Increased relaxation / calmness; less restlessness.
2) Medium Quality	2) Nurses (90%); persons employed in pastoral care, respiratory therapy and social work	2) TG: n = 12 (T2) / CG: n = 13 (T2); waiting-list control / Drop-outs: n = 2	Qualitative data: Weekly and final evaluation forms during the program and on the last night of the program: *questions about the results and importance of the MBSR program*	Improvement of self-care.
	3) Mean age TG and CG: 46; TG: unknown; CG: unknown	3) Moments of measurement: 1. at baseline (T1); 2. post class (T2); 3. 3-month post-intervention (T3)	Qualitative data: E-mails (received from 7 participants) during the program and several months afterwards	Better work and family relationships.
	4) % male: 0%	4) MBSR program: 8 weekly 2.5 h sessions; daily home practice, 6 days per week; daylong retreat	Qualitative data: 2 types of depth Interviews: first with 4 graduates; second with the Vice President for Clinical Services, the fourth author, about her motivation for supporting and her impression of the results.	Improvement of dealing with difficult emotions (mood / resilience).
			Qualitative data: Focus group (7 graduates) about changes	A decrease of physical pain.
1) Martín Asuero et al. [90]	1) N = 68	1) Quasi randomized controlled study	1) Maslach Burnout Inventory (MBI) (2)	Significant reduction of emotional exhaustion and depersonalization and total in the TG compared to the CG. A significant increase of personal accomplishment in the TG. (2)
2) Medium Quality	2) Primary health care professionals: physicians (60%); nurses (33.3%); social workers and clinical psychologists (6,7%)	2) TG: n = 43 / CG: n = 25 / Drop-outs: n = 0	2) Profile of Mood States (POMS), short version. Five subscales: tension-anxiety; depression-dejection; anger-hostility; vigor-activity; fatigue-inertia (5; 12)	Significant reduction in the TG in total mood disturbance and in the following subscales: depression, anger, tension and fatigue; no significant change in the vigor scale. Significant reduction in the TG compared to the CG: total mood disturbance; tension and fatigue. (5; 12)
	3) Mean age TG and CG: 47	3) Moments of measurement: 1. at baseline; 2. post class	3) Jefferson Scale of Physician Empathy. Three subscales: compassionate care; perspective taking; “standing in the patient’s shoes”	Significant increase of compassionate care in the TG. A significant increase in empathy and “standing in the patient’s shoes” in the TG compared to the CG.
	4) % male: 8%	4) MBSR program: 8 weekly 2.5 h sessions; daily home practice, 6 days per week; daylong retreat of 8 hours	4) Five Facets Mindfulness Questionnaire (FFMQ) (1)	Significant increase in the TG in all subscales except for describing. Significant increase in observing, non-reactivity and total in the TG compared to the CG. (1)
			5) Questionnaire on changes in personal habits and mindfulness practice	All participants in the intervention group reported feeling better after the intervention.
1) Vega et al. [91]	1) N = 103	1) Controlled study	1) Attentional measure: Continuous Performance Test (CPT)	1) No important significant changes.
2) Medium Quality	2) Psychotherapists: psychiatrists and clinical psychologists	2) TG: n = 58 / CG: n = 43; waiting-list control / Drop-outs: n = 2	2) Attentional measure: Stroop Task	2) Significant increase in attentional control (specifically, task switching) in the TG compared to the CG.
	3) Mean age TG: 29.6; CG: 28.4	3) Moments of measurement: 1. at baseline; 2. post class	3) Emotional measure: State-Trait Anxiety Inventory (STAI) (9)	3) Significant decrease in anxiety state in the TG compared to the CG. (9)
	4) % male: 26%	4) MBSR program: 8 weekly 2.5 h sessions	4) Emotional measure: State-Trait Anger Expression Inventory-2 (STAXI-2); many subscales	4) Significant decrease in Angry Reaction subscale in the TG compared to the CG.
			5) Emotional measure: Beck Depression Inventory (BDI) (5)	5) Significant decrease in depression scores in the TG compared to the CG (limited clinical significance). (5)
			6) Mindfulness scale: Attention Awareness scale (MAAS) (1)	6) Significant increase in mindfulness in the TG compared to the CG. (1)
1) Frank et al. [85]	1) N = 36	1) Quasi randomized controlled study	1) Brief Symptom Inventory (BSI) (psychological distress) with the following subscales: anxiety; somatization; depression; Global Severity Index (GSI) (4)	No significant changes in anxiety, somatization, depression and general symptoms were revealed. (4)
2) Medium Quality	2) High school educators: full time employed; 94.4% had completed 18 or more years of education	2) TG: n = 18 / CG: n = 18, waiting-list control / Drop-outs: n = 0	2) Pittsburgh Sleep Quality Index (PSQI): general sleep quality; sleep latency; sleep duration; habitual sleep efficiency; sleep disturbances; the use of medication to sleep; daytime sleep-related dysfunction over the past month (7)	Significant improvements in all aspects, except for sleep efficiency and the use of medication to sleep, in the TG compared to the CG. (7)
	3) Mean age TG and CG: 40.72	3) Moments of measurement: 1. at baseline; 2. post class	3) Self-compassion Scale (SCS). Subscales (self-kindness; self-judging; common humanity; isolation; mindfulness; over-identification) and total self-compassion (6)	Significant improvements in all subscales, except for common humanity and isolation, in the TG compared to the CG. (6)
	4) % male: 22.2%	4) MBSR program: 8 weekly 2 h sessions; 25–30 min daily home practice, 6 days per week	4) Maslach Burnout Inventory (MBI) (2)	No significant changes. (2)
			5) Five Facets Mindfulness Questionnaire (FFMQ) (1)	Significant improvements in all subscales, except for describing, in the TG compared to the CG. (1)
			6) Affective Self-Regulatory Efficacy Scale (ASRES). Subscales: calmness; acknowledgement; present moment; acceptance (13)	Improvements in all subscales, except for acceptance, in the TG compared to the CG. (13)
1) Jennings et al. [83]	1) N = 53	1) Randomized controlled study	1) General well-being scale: Positive and Negative Affect Schedule (PANAS): positive and negative affect subscales (12)	No significant changes. (12)
2) Medium Quality	2) Teachers in (sub)urban public schools: 72% had a graduate degree; average years of teaching, 11.7; 47% taught at the elementary level; the remaining teachers taught at the preschool, middle or high school; or in mixed grade settings	2) TG: n = 25 / CG: n = 25, waiting-list control / Drop-outs: n = 3	2) General well-being scale: Emotion Regulation Questionnaire (ERQ). Two subscales: cognitive reappraisal; expressive suppression	Significant increase in cognitive reappraisal in the TG compared to the CG.
	3) Mean age TG and CG: 36	3) Moments of measurement: 1. at baseline; 2. post class	3) General well-being scale: Center for Epidemiologic Studies Depression Scale (CES-D-20) (5)	No significant changes. (5)
	4) % male: 11%	4) Modified MBSR program (CARE): 30-hr program in 4 day-long sessions over 4–6 weeks; intersession 20-to-30-min phone coaching; 1-day booster two months later	4) General well-being scale: Daily Physical Symptoms (DPS)	Significant decrease in daily symptoms in TG compared to the CG.
			5) Teachers’ Sense of Efficacy Questionnaire (TSES). Total score and three subscales: instructional strategies; student engagement; classroom management (14)	Significant increase in total sense of self-efficacy, instructional strategies and student engagement in the TG compared to the CG. (14)
			6) Maslach Burnout Inventory (MBI) (2)	Significant increase in personal accomplishment in the TG compared to the CG. (2)
			7) Time Urgency Scale (TUS). A total scale score and 5 subscales: speech patterns; eating behavior; task-related hurry; general hurry; competitiveness	Significant improvement in general hurry in the TG compared to the CG.
			8) Five Facets Mindfulness Questionnaire (FFMQ) (1)	Significant increase in the subscales observing, non-reactivity and in the total mindfulness score in the TG compared to the CG. (1)
			9) Program evaluation: Care Acceptability Questionnaire (CAQ)	87% of the teachers (strongly) agreed.
1) Leroy et al. [9]	1) N = 90	1) Controlled study	1) Mindfulness Attention Awareness scale (MAAS) (1)	A significant increase in mindfulness for the group as a whole between T1 and T2; the increase in TG is however significantly higher. (1)
2) Medium Quality	2) Employees in the area of telecommunication, consulting and architecture (for profit) and parliamentary services, public services and health insurance (not-for-profit)	2) TG: n = 76 (before drop-out!) (six groups); TG after drop out unknown; CG: n = 14 (before drop-out!) (two groups), waiting-list control; CG after drop out unknown / Drop-outs at T1: 7 of 90; at T2: 14 of 90; at T3: 22 of 90	2) Authentic functioning index of Leroy et al (in a work related setting)	A significant increase in authentic functioning for the group as a whole between T1 and T2; the increase in TG is however significantly higher. Authentic functioning mediates the relationship between mindfulness and work engagement, partially for the static relationship (at one specific point in time) and fully for the dynamic relationship (different points in time).
	3) Mean age TG and CG: 42; TG: unknown; CG: unknown	3) Moments of measurement: 1. before training (T1); 2. 2 month post-intervention (T2); 3. 4 month post-intervention (T3)	3) Measure of work engagement (15)	A significant increase in work engagement for the group as a whole between T1 and T2; the increase in TG is however significantly higher. (15)
	4) % male: ± 25%	4) MBSR: 8 weekly 3 h sessions; exercise at home or at work	4) Amount of days meditating each week (at T2 and T3) (control variable)	A significant interaction effect between time and meditation practice during training in TG. The amount of meditation practice has a significant positive effect on mindfulness and authentic functioning but not on work engagement.
1) Manotas et al. [80]	1) N = 131	1) Randomized controlled study	1) Five Facets Mindfulness Questionnaire (FFMQ) (1)	Significant increase in observing, non-judging and in the total mindfulness score in the TG compared to the CG. (1)
2) Medium Quality	2) Colombian health care professionals: medical doctors (16.9%); nurses (45.8%); scientists (14.5%); other helping professionals (18.1%); 4 participants without employment data (4.7%)	2) TG: n = 40 (26 drop-outs) / CG: n = 43 (22 drop-outs); waiting-list control / Drop-outs: 48	2) Brief Symptom Inventory (BSI) (psychological distress) (4)	Significant decrease in the GSI and in the three subscales for the TG compared to the CG. (4)
	3) Mean age TG and CG: 39.05	3) Moments of measurement: 1. at baseline; 2. post class	3) Perceived Stress Scale (PSS-14) (3)	Significant decrease in perceived stress for the TG compared to the CG. (3)
	4) % male: 9.6%	4) Shortened MBSR program: 4 weekly 2h sessions; daily homework		
1) Mackenzie et al. [79]	1) N = 30	1) Randomized controlled study	1) Maslach Burnout Inventory (MBI) (2)	Significant reduction of emotional exhaustion in the TG compared to the CG. Depersonalization remains stable in the TG and increases significantly in the CG. A significant increase of personal accomplishment in the TG compared to the CG. (2)
2) Medium Quality	2) Nurses and nurse aides	2) TG: n = 16 / CG: n = 14, waiting-list control / Drop-outs: n = 0	2) The Smith Relaxation Dispositions Inventory (8)	Significant increases in relaxation in the TG compared to the CG. (8)
	3) Mean age TG: 48.62; CG: 44.78	3) Moments of measurement: 1. at baseline; 2. post class	3) The Intrinsic Job Satisfaction subscale from the Job Satisfaction Scale	A clear, although not significant improvement (*p* = 0.06), in the TG
	4) % male: 3%	4) Shortened MBSR program:4 weekly 30 min sessions; daily at least 10 minutes exercise, 5 days per week	4) The Satisfaction with Life Scale (12)	Significant positive changes in life satisfaction in the TG compared to the CG. (12)
			5) The 13-item version of Antonovsky’s Orientation to Life Questionnaire (Sense Of Coherence: the ability to view life as meaningful, comprehensible and manageable)	The sense of coherence doesn’t improve more in the TG than in the CG.
1) Shapiro et al. [53]	1) N = 38	1) Randomized controlled study	1) Maslach Burnout Inventory (MBI) (2)	No significant reduction of job burnout in the TG compared to the CG. (2)
2) Medium Quality	2) Health-care professionals: physicians, nurses, social workers, physical therapists, psychologists	2) TG: n = 10 (8 drop-outs) / CG: n = 18 (2 drop-outs), waiting-list control / Drop-outs: n = 10	2) Perceived Stress Scale (3)	A significant stress reduction in the TG compared to the CG. (3)
	3) Mean age TG: unknown; CG: unknown	3) Moments of measurement: 1. at baseline; 2. post class	3) Satisfaction With Life Scale (SWLS) (11)	Clear, although not significant (*p* = 0.06), improvements in the TG compared to the CG. (11).
	4) % male: unknown	4) MBSR program: 8 weekly 2 h sessions; daily home practice, 6 days per week; daylong retreat	4) Self-compassion Scale (6)	A significant increase in self-compassion in the TG compared to the CG. (6)
			5) Brief Symptom Inventory (BSI)(psychological distress) (4)	No significant decrease in the TG compared to the CG. (4)
			6) Open ended question (qualitative data)	
1) Klatt et al. [84]	1) N = unknown	1) Randomized controlled study	1) Connor-Davidson Resiliency Scale (CD-RISC), 10-items version	Significant increase in resilience in the TG (compared to the CG?)
2) Low Quality	2) Employees of Intensive Care Units	2) TG: n = 34 / CG: n = unknown, waiting-list control / Drop-outs: unknown	2) Utrecht Work Engagement Scale (UWES), 9-items version. Subscales: vigor; dedication; absorption (15)	Significant increase in work engagement in the TG (compared to the CG?) (mostly induced by the vigor subscale). (15)
	3) Mean age TG and CG: unknown	3) Moments of measurement: 1. at baseline; 2. post class	3) Number of Breaths/30 sec (self-measured) at the beginning and the end of each session	Significant decrease in the pre-post breath counts in weeks 1–3, 5–6 and week 8 of the intervention in the TG.
	4) % male: unknown	4) Modified MBSR program (Mindfulness In Motion): 8 weekly 1 h sessions; 20 min daily home practice, at least 5 days per week; 2 h ‘retreat’	4) Program evaluation	Highly valued.
1) Beshai et al. [75]	1) N = 89	1) Non-randomized study	1) Perceived Stress Scale (PSS) (3)	Significant reduction in perceived stress in the TG compared to the CG. (3)
2) Low Quality	2) Secondary school teachers and staff	2) TG: n = 49 / CG: n = 40, waiting-list control / Drop-outs: n = 0	2) Warwick-Edinburgh Mental Well-being Scale (WEMWBS) (11)	Significant increase in well-being in the TG compared to the CG. (11)
	3) Mean age TG: unknown; CG: unknown	3) Moments of measurement: 1. at baseline; 2. post class	3) Five Facets Mindfulness Questionnaire (FFMQ) (1)	Significant increase in mindfulness in the TG compared to the CG. (1)
	4) % male: 30.34%	4) Modified MBSR program with aspects of MBCT: 9 sessions during 8 weeks: a presentation and eight 75 min sessions; 10-40-minute home practice, 6 days per week	4) Neff Self-Compassion Scale (SCS), using two of the six subscales: self-judgment and self-kindness. Self-compassion: the two subscales combined (6)	Significant increase in self-compassion in the TG compared to the CG. (6)
			5) Teachers Feedback: acceptability / enjoyment and learning	95% of the participants who attended the course found it to be acceptable.
1) Geary and Rosenthal [86]	1) N = 108	1) Between group; quasi random	1) Cohen’s Perceived Stress Scale (PSS) (3)	A significant stress reduction in the TG compared to the CG between T1 and T2, and between T1 and T3. (3)
2) Low Quality	2) Academic healthcare employees or relatives of employees	2) TG: n = 59 / CG: n = 49 / Drop-outs: n = 0	2) SCL-90-R (psychological distress) (4)	A significant reduction in psychological distress in the TG compared to the CG between T1 and T2, and between T1 and T3. (4)
	3) Mean age TG: 48; CG: 42	3) Moments of measurement: 1. at baseline; 2. post class; 3. 1-year post-intervention measurement	3) SF-36 Measure of Health and Well-Being (11)	Significant positive changes in life satisfaction or general wellbeing in the TG compared to the CG between T1 and T2, and between T1 and T3. (11)
	4) % male: 8%	4) MBSR program: 8 weeks program; class meeting each week 3 hours; between class 5 and class 7 an 8-hour retreat	4) Daily Spiritual Experiences Scale DSES)	A significant increase of daily spiritual experiences in the TG compared to the CG between T1 and T2, and between T1 and T3.
1) Poulin et al. [8]	1) N = 40	1) Between group; quasi random	1) Maslach Burnout Inventory (MBI) (2)	A significant reduction of emotional exhaustion in the TG compared to the CG. (2)
2) Low Quality	2) Nurses or nurse aides	2) TG: n = 16; brief MBSR / CG bIPMR: n = 10 / CG 2 (no intervention): n = 14 / Drop-outs: n = 0	2) Satisfaction With Life Scale (SWLS) (11)	Significant positive changes in life satisfaction or general well-being in the TG compared to the CG. (11)
	3) Mean age TG: 48.6; CG bIPMR (brief Imagery and Progressive Muscle Relaxation): 46.0; CG 2 (no intervention): 44.8	3) Moments of measurement: 1. at baseline; 2. post class	3) Smith Relaxation Disposition Inventory (SRDI) (8)	Significant changes in relaxation in the TG compared to the CG. (8)
	4) % male: 5%	4) Brief MBSR program: four 30-minute training sessions; home practice 15 to 20 minutes per day. The control bIPMR: matched to the bMBSR intervention (length; the balance of didactic and experiential focus, homework and support material)		
1) Walach et al. [92]	1) N = 29	1) Between group; quasi random	1) Interviews (qualitative)	**Qualitative data**: increased awareness of work-related problems contributing to stress; more critical toward the work environment
2) Low Quality	2) Workers in a service center	2) TG: n = 11 (T2; T3) / CG: n = 16 (T2; T3); waiting-list control / Drop-outs: n = 2	2) Coping with stress (SVF 120, Germany)	Significant increase of positive coping strategies in the TG compared to the CG between T1 and T2; no significant group differences for negative coping strategies
	3) Mean age TG: 41.3; CG: 33.7	3) Moments of measurement: 1. at baseline (T1); 2. post class (T2); 3. 2-month post-intervention measurement (T3)	3) SALSA (Salutogenetic Subjective Analysis of the Workplace): used one part of the battery covering job characteristics; job demand and stress; organizational resources; social resources in the work place	No significant changes
	4) % male: 41%	4) MBSR program: 8 weekly 2,5 h sessions; 30 min daily home practice, 6 days per week; daylong retreat	4) Locus of control (the Fragebogen zu Kontrollüber-zeugungen: FKK) (14)	No significant changes. (14)
			5) Freiburg Complaint List ((FBL): subscales General Complaints; Tension; Tiredness (4)	No significant changes. (4)
			6) Satisfaction with Life (the Fragebogen zur Lebenszufriedenheit: FLZ): subscales Health, Financial Satisfaction, Leisure, Own Person, and Friends and Social Relations (11)	No significant changes. (11)
1) Horner et al. [77]	1) N = 74	1) Quasi randomized controlled study	1) Professional Quality of Life (ProQOL) Scale Version 5^c.^ Two subscales: compassion satisfaction; burnout (11)	No significant changes. (11)
2) Low Quality	2) Workers in two medical-surgical units: staff nurses; nurse aides; clinical secretaries; unit manager; supervisor	2) TG: n = 31 (15 drop-outs) / CG: n = 12 (16 drop-outs) / Drop-outs: n = 31	2) Mindful Attention Awareness Scale (MAAS) (1)	No significant changes. (1)
	3) Mean age TG and CG: unknown	3) Moments of measurement: 1. at baseline; 2. post class	3) Self-reports of individual and unit stress levels (3)	No significant changes. (3)
	4) % male: unknown (primarily female)	4) Shortened MBSR program: weekly 30 min sessions	4) Hospital Consumer Assessment of Healthcare Providers and Systems (HCAHPS)	Patient satisfaction scores in the TG increased on ‘overall rating’ and ‘communication with nurses’. No significant changes.

^1^ Number corresponds with the results in the chapter: *The effects of MBSR on employees’ mental health*

### Effects of mindfulness interventions on patients

Previous research on the effects of group-based mindfulness interventions has focused on benefits for various patients groups (e.g., those with chronic pain, anxiety, eating and major depressive disorders, fibromyalgia, psoriasis, or cancer [29]). These studies found that mindfulness decreases stress sensitivity [30–33], increases stress management [34], improves concentration [35], improves physical resilience [36–38], and reduces symptoms of anxiety and depression [39–41]. More recent studies have reported positive effects of mindfulness interventions on chronic pain [42], immunity [43], generalized anxiety disorders [44], eating disorders [45], depression relapse [21, 46], addiction [47], and fibromyalgia [48, 49]. In their meta-analysis, Strauss et al. [50] identified potential benefits for depression, but not for anxiety disorders.

### Effects of mindfulness interventions on healthy people and professionals

Following on from the promising results for patients, MBSR and MBCT have recently also been used for healthy people [51] and for employees and managers in a healthcare setting [52]. A meta-analysis by Chiesa and Serretti [51] focusing on healthy participants (not explicitly on employees) identified ten studies on MBSR, most of them of low methodological quality. The most striking outcome was the reduction of stress levels.

Employees and managers in a healthcare setting are regularly confronted with stress in the form of physical and mental suffering as well as strong emotions (their own or those of their patients) [53]. Adequate stress management can improve the health of these professionals [54] and the quality of care they provide to patients [55].

Mindfulness training is assumed to also have potential for other demanding work environments, as a means of improving employees’ health and work engagement and, consequently, the quality of services provided to clients. To date, however, there has been no systematic review of studies investigating the effects of mindfulness training on the mental health of employees across different occupational sectors.

The most commonly studied group of employees are healthcare professionals: four reviews [52, 56–58] and two reviews/meta-analyses [59, 60] focused exclusively on healthcare professionals and students. Escuriex and Labbé [56] found no clear correlation between a therapist’s mindfulness and treatment outcomes. Irving et al. [52] reported that MBSR benefits the physical and mental health of clinicians. Morgan et al. [57] discussed 14 qualitative studies and concluded that the benefits of MBSR “ranged from increased personal well-being and self-compassion to enhanced presence when relating to others, leading to enhanced compassion and a sense of shared humanity” (p. 744). Smith [58], who examined nurses, concluded that MBSR helps them deal with work-related stress. The meta-analysis by Regehr et al. [59] supports the idea that mindfulness interventions reduce stress, anxiety, and burnout among medical students and practicing physicians. Similarly, Burton et al.’s [60] meta-analysis suggests that mindfulness training can significantly decrease stress levels among healthcare professionals.

Virgili [61] performed a meta-analysis involving three subgroups of employees: healthcare professionals, teachers, and general/office employees. The aim was to examine the effect of a mindfulness-based intervention on a single outcome: employees’ psychological distress. Unfortunately, the composition of the outcome ‘psychological distress’ was not explained and no other outcomes, positive or negative, were considered [61].

Two other meta-analyses [51, 62] did not explicitly focus on employees. Grossman et al. [62] examined a relatively small number of clinical and stressed non-clinical participants and found possible benefits of MBSR. Finally, another recent review [63] provided strong evidence that mindfulness-based interventions can reduce occupational burnout among healthcare professionals and teachers.

### Aim of the study

The aim of this systematic review is to gain deeper insight into the effects of two mindfulness interventions—MBSR and MBCT training—on employees’ mental health across different occupational sectors. Looking at different occupational sectors gives us an opportunity to draw general conclusions about the effects of the interventions on employees and to identify potential differences between sectors.

This review uses the World Health Organization’s (WHO) definition of mental health as “a state of well-being in which the individual realizes his or her own abilities, can cope with the normal stresses of life, can work productively and fruitfully, and is able to make a contribution to his or her community” [64]. The WHO definition has three main components: emotional or subjective well-being [65], psychological well-being (referring to optimal functioning in work and life) [66], and social well-being (targeting optimal functioning in social groups and society) [67].

## Method

**Fig 1:**
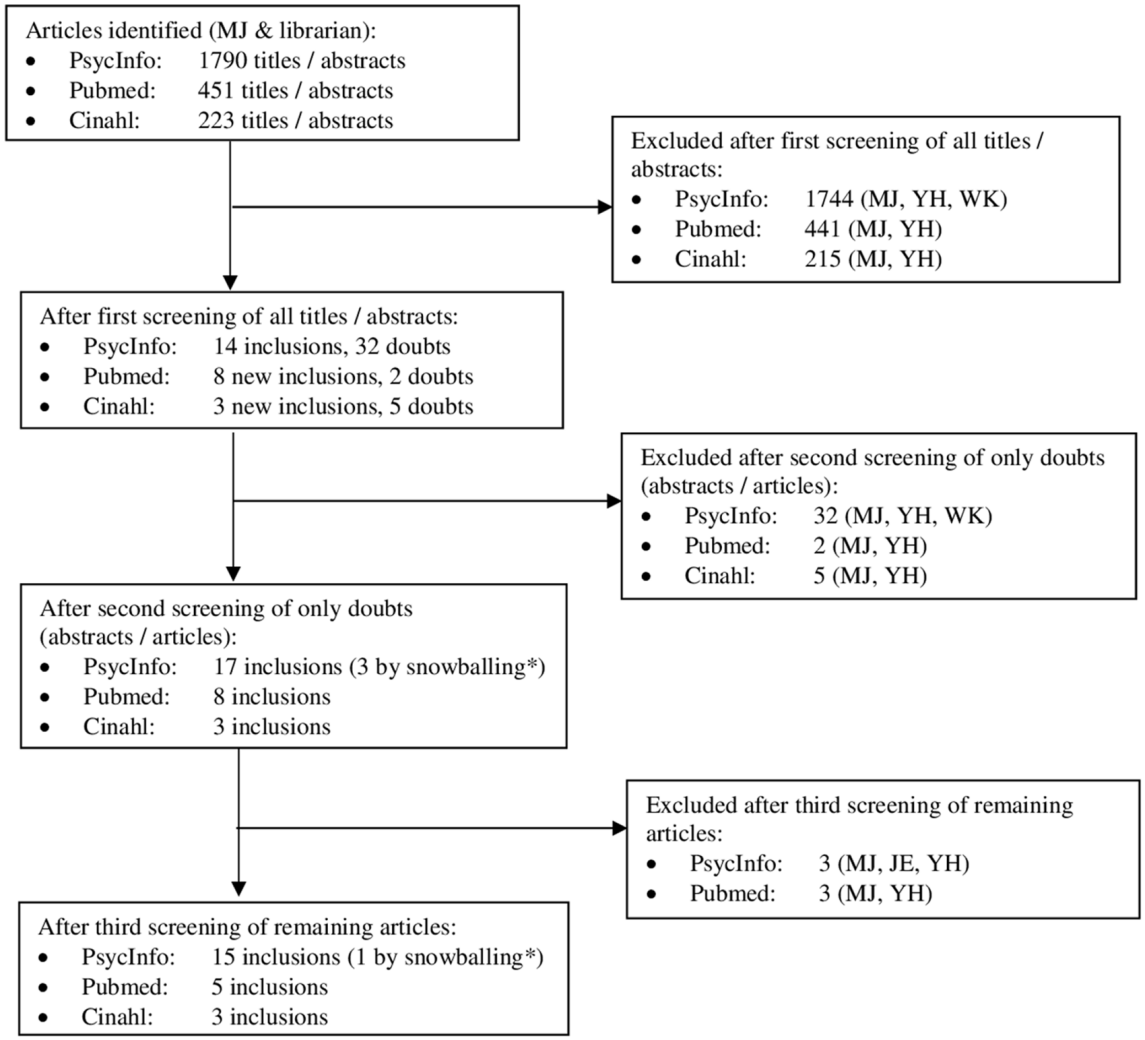
Flow chart showing the review process and results. * Once we had compiled a set of eligible studies, we searched their reference lists for additional studies (‘snowballing’ or ‘snowball’ method).

We conducted a systematic search of the literature with three rounds of screening. The flow chart in Fig 1 outlines our review process and findings.

### Search strategy

We searched three electronic databases (PsycINFO, PubMed, and CINAHL) for scientific studies examining the efficacy of MBSR and MBCT on employees’ mental health and well-being. We used the following search string in each database: (Employees OR workers OR managers OR professionals OR work OR labour OR labor OR job* OR employ* OR vocational*) AND (mindfulness OR mbsr OR mbct).

### Search

All searches were performed in October 2015. The search in PsycINFO yielded 1790 articles. The following procedure was used to screen them:

1. First screening of the titles/abstracts. Independent categorization (include, exclude, or unclear) by three authors, as follows:
  - The first author (MJ) screened all 1790 articles based on the titles and abstracts.
  - To check the quality of this screening, the second (YH) and third (WK) authors independently screened random samples from the 1790 articles; specifically, from every five articles, one abstract was randomly selected and screened. Any differences in opinion among the three authors were discussed until consensus was reached.
2. Second screening of articles whose relevance was classed as ‘unclear’. All 32 articles were independently screened by the first three authors as follows:
  - Each author assessed each abstract and, if necessary, the entire article.
  - Any differences in categorization were discussed until consensus was reached.
3. Third screening of all remaining articles. The first, second, and fifth authors (MJ/YH/JE) assessed the full texts of the 18 articles initially labelled ‘include’ as follows:
  - Each author independently assessed the articles.
  - They then discussed each article until consensus was reached as to their in-/exclusion.

The same search string and screening procedure were used for the 451 and 223 articles obtained from PubMed and CINAHL, respectively.

### Inclusion and exclusion criteria

The following inclusion criteria were used for articles in the first and second screenings:

- Full text was available.
- Published in English.
- Mindfulness training (MBSR or MBCT) was operationalized as “moment to moment awareness to be cultivated with a nonjudgmental attitude; teaching of formal meditation techniques; and stressing the importance of daily and systematic practice” [16]. Once we had compiled a set of eligible studies, we searched their reference lists for additional relevant studies (‘snowball’ method). For articles that did not provide a definition of mindfulness training, two criteria had to be met:
  - References to relevant authors (e.g., Jon Kabat-Zinn, Ruth Baer, Mark Williams, John Teasdale, Zindel Segal).
  - Presence of the following elements in the mindfulness training: a focus on the ‘here and now’, an emphasis on increasing consciousness, learning to use a nonjudgmental attitude, learning formal meditation techniques, and daily and systematic practice.
- Study population: employees or managers in workplace settings.
- Group-based rather than individual interventions (i.e., no individual therapy or coaching).
- At least four face-to-face 30-minute sessions.
- Qualitative and/or quantitative work-related outcomes, including psychological/mental factors.

The following exclusion criteria was used in the first and second screenings:

- Dissertations, conference papers, reviews, chapters in handbooks, editorials.
- Studies on forms of meditation that do not emphasize mindfulness, such as transcendental meditation. In some meditation approaches, attention is directed towards one object or stimulus, such as a word (a mantra), sound, or sensation. In contrast, mindfulness meditation emphasizes the observation of constantly changing internal and external stimuli [29].
- Studies on the cognitive model of mindfulness developed by Ellen Langer [68, 69], which involves working with external material such as information and includes active, goal-oriented cognitive tasks, such as problem solving. We consider her approach to be distinct from that used by other mindfulness studies.
- Studies on interventions that included mindfulness training as a component of a broader treatment program, such as Dialectical Behavior Therapy [26], Acceptance and Commitment Therapy [27], and Mindfulness-Based Relapse Prevention [28].
- Population: employees with a specific mental or physical disorder and students/trainees.
- Pre-post design (i.e., without a control group) (see e.g. Bazarko et al. [70]).

The inclusion criteria for the third screening were:

- Description of qualitative and/or quantitative psychological/mental outcomes.
- Research design:
  - Randomized controlled trial (RCT), or
  - Quasi-experimental design with an intervention group and a control group, where the control group comprised those on a waiting list or those receiving treatment as usual (e.g., relaxation practices, Jacobson relaxation, or an attention placebo) (quasi-RCT).

### Methodological quality and levels of evidence

As indicated above, we included two types of research design—RCT and quasi-RCT—and excluded pre-post studies. In RCT and quasi-RCT studies, participants in the control group (CG) were compared with those in the treatment group (TG) both before and after the treatment (T1 vs. T2, between-group analysis).

The methodological quality of the selected studies was assessed using the nine criteria outlined in a paper entitled ‘Assessment of a randomized controlled trial’ by the Dutch public health institute Kwaliteitsinstituut voor de Gezondheidszorg CBO [71]. The studies were classified as ‘high quality’ if they met ≥ 7 of the criteria, as ‘medium quality’ if they met 5 or 6 of the criteria, and as ‘low quality’ if they met ≤ 4 of the criteria.

The nine criteria were formulated as closed questions with three possible answers: (1) yes, (2) no, or (3) not enough information. The questions were as follows:

1. Were the participants randomly assigned to the intervention?
2. Was the researcher (or research assistant) who assigned the participants blinded to the randomization order?
3. Were the participants blinded to the treatment?
4. Were the practitioners blinded to the treatment?
5. Were the assessors who evaluated the effects (i.e., mental health outcomes) of the treatment blinded to the treatment?
6. Were the control and intervention groups comparable at the beginning of the trial?
7. Were enough participants available for follow-up after the intervention(s)?
8. Were the participants analyzed in the group in which they were randomized?
9. Were the groups treated in the same manner, except for the intervention(s)?

Domusmedica [72] outlines three levels for assessing the importance of outcomes. An outcome can be classified as Level 1 (‘there is evidence for …’ / ‘it has been proven that …’) if at least two high-quality RCTs show significance between groups (change in TG versus change in CG). An outcome can be classified as Level 2 (‘it is plausible that …’) if at least two medium-quality RCTs show significance between groups. Finally, an outcome can be classified as Level 3 (‘there are indications that …’) if at least one medium-quality RCT shows significance.

We used five levels of evidence based on the levels described above. Our definitions of Levels 1 and 2 were the same as the Domusmedica definitions, although we found no outcome that could be classified as Level 1 [72]. Our definition of Level 3 was slightly different: we classified an outcome as Level 3 if at least one medium-quality RCT or three low-quality experiments (RCT, quasi-RCT) showed significance between groups (change in TG versus change in CG). Level 4 (‘the evidence is weak’) outcomes refer to one or two low-quality experiments (RCT, quasi-RCT) with significance between groups (change in TG versus change in CG). Level 5 (‘there is no evidence’) indicates that there are no studies with significant results between groups for the relevant outcome.

## Results

### Studies included

The results of the review process are shown in Fig 1. The PsycINFO search and three screenings yielded 16 articles. The PubMed and CINAHL searches and screenings resulted in five and three additional articles, respectively. We thus assessed 24 articles in total, describing 23 studies.

### Characteristics of studies

Table 1 presents an overview of the 24 articles, including the number of participants and their characteristics, the research design, treatment method, assessment instruments, and outcomes. The 24 articles refer to 23 studies as two articles described the same study, presenting the quantitative [73] and qualitative [74] data separately.

All studies primarily focused on MBSR. One study used MBSR in combination with some aspects of MBCT [75]. As this modified MBSR program was developed for healthy rather than depressive teachers, this study was nevertheless included. The MBSR program of the other 22 studies varied: 1 used an extended MBSR program [7], 8 used a shortened MBSR program [8, 76–82], 2 studies also used a modified MBSR program [83, 84], and the other 11 used the usual MBSR program [9, 53, 73, 85–92]. As no study concentrated exclusively on MBCT, we will not address MBCT in the rest of this paper.

Of the 23 studies, 13 were RCTs [7, 53, 73, 76, 78–82, 84, 87–89] and 10 were quasi-RCTs [8, 9, 75, 77, 83, 85, 86, 90–92]. Three [77, 86, 90] had no intervention for the control group. One study [8] had two control groups: one with an additional treatment and one with no intervention. Another study had three treatment groups and a control group with no intervention [82]. The other 18 studies [7, 9, 53, 73, 75, 76, 78–81, 83–85, 87–89, 91, 92] had a ‘waiting-list control’ (i.e., they did not include an additional treatment intervention to control for factors such as trainer support, group support, and home practice).

Sample sizes and composition also varied. The sample sizes in 11 studies [7, 8, 53, 73, 76–79, 81, 85, 92] were relatively small: the group sizes (TG and CG) ranged from 10 to 24 participants. The most frequently studied research populations were healthcare professionals (12 studies) [7, 53, 73, 76, 77, 79–81, 84, 86, 90, 91] (e.g., nurses, nurse aides, nursing leaders, physicians, social workers, psychologists, psychotherapists, physical therapists, psychiatrists) and teachers (five studies) [75, 83, 85, 88, 89]. Five studies [7, 8, 79, 81, 85] involved participants with the same occupation; four studies [83, 85, 88, 89] investigated respondents from related occupations (i.e., elementary, secondary, or high school teachers). In the other studies, the participants’ occupations varied.

Participants’ personal characteristics were also diverse. Twenty of the 23 studies included more female than male participants; only Huang et al. [87] reported more male participants, while the other two studies did not indicate the gender of the participants [53, 84]. The mean age of participants was over 40 years in 15 studies [7–9, 73, 76, 78, 79, 81, 82, 85–90], less than 40 years in four studies [80, 83, 91, 92], and not stated in four studies [53, 75, 77, 84]. Twenty studies were primarily quantitative, and only three contained a substantial amount of qualitative data [73, 89, 92].

### Quality of the studies

Table 2 shows the classification of the studies as ‘high quality,’ ‘medium quality,’ or ‘low quality.’ Two studies were of high quality [76, 87], 15 were of medium quality [7, 9, 53, 73, 78–83, 85, 88–91], and six were of low quality [8, 75, 77, 84, 86, 92].

**Table 2:** Quality criteria and quality of the selected studies quality criteria. 1) Assignment intervention randomized; 2) Includer blinded for randomization order; 3) Employees blinded for treatment; 4) Practitioner blinded for treatment; 5) Assessor blinded for treatment (mental health outcomes); 6) Groups comparable at the start of the trial; 7) Follow up available of enough included employees at T1, etc. ^l^; 8) Included employees analyzed in randomized group; 9) Same treatment of groups except the intervention.

Quality criteria	1	2	3	4	5	6	7	8	9	Total quality score	Quality label
Studies											
Duchemin et al. [76]	+	+	-	-	+	+	+	+	+	7	HQ
Huang et al. [87]	+	+	-	-	+	+^a^	+	+	+	7	HQ
Amutio et al. [7]	+	+	-	-	+	?	+	+	+	6	MQ
Taylor et al. [89]	+	?	-	-	+^b^	+^c1^	+	+	+	6	MQ
Roeser et al. [88]	+	?	-	-	+	+^c2^	+	+	+	6	MQ
Wolever et al. [82]	**+**	**?**	**-**	**-**	**+**	**+**	**+**	**+**	**+**	6	MQ
Pipe et al. [81]	**+**	**?**	**-**	**-**	**+**	**+**	**+**	**+**	**+**	6	MQ
Klatt et al. [78]	**+**	**?**	**-**	**-**	**+**	**+**	**+**	**+**	**+**	6	MQ
Cohen-Katz et al. Part II [73]; Cohen-Katz et al. Part III [74]	**+**	**?**	**-**	**-**	+^b^	**+**^d^	**+**	**+**	**+**	6	MQ
Martín-Asuero et al. [90]	- ^e^	-	-	-	+	+	+	+	+	5	MQ
Vega et al. [91]	-	-	-	-	+	+	+	+	+	5	MQ
Frank et al. [85]	- ^e^	-	-	-	+	+	+	+	+	5	MQ
Jennings et al. [83]	+	?	-	-	+	+	?	+	+	5	MQ
Leroy et al. [9]	**-**	**?**	**-**	**-**	**+**	**+**	**+**	**+**	**+**	5	MQ
Manotas et al. [80]	+	?	-	-	+	+	-	+	+	5	MQ
Mackenzie et al. [79]	**-** ^f^	**?**	**-**	**-**	**+**	**+**^g^	**+**	**+**	**+**	5	MQ
Shapiro et al. [53]	**+**	**?**	**-**	**-**	**+**	**+**^h^	**-** ^i^	**+**	**+**	5	MQ
Klatt et al. [84]	+	?	-	-	+	?	?	+	+	4	LQ
Beshai et al. [75]	-	-	-	-	+	-	+	+	+	4	LQ
Geary and Rosenthal [86]	**-** ^e^	**-**	**-**	**-**	**+**	**-**^j^	**+**	**+**	**+**	4	LQ
Poulin et al. [8]	**-** ^e^	**-**	**-**	**-**	**+**	**-**^k^	**+**	**+**	**+**	4	LQ
Walach et al. [92]	**-** ^e^	**-**	**-**	**-**	**+**	**-**^g^	**+**	**+**	**+**	4	LQ
Horner et al. [77]	- ^e^	-	-	-	+	?	-	+	+	3	LQ

HQ = high quality (meets ≥ 7 of the criteria), MQ = medium quality (meets 5 or 6 of the criteria), LQ = low quality (meets ≤ 4 of the criteria).

Criteria are based on an assessment guide titled ‘assessment of a randomized controlled trial’ by the Dutch public health institute ‘Kwaliteitsinstituut voor de Gezondheidszorg CBO’ [71].

^a^: except gender

^b^: not for the interviews

^c1^: except occupational stress;

^c2^: except burnout

^d^: unknown for age

^e^: quasi random

^f^: ‘Because the study was conducted during the summer, however, several exceptions were made ……’ (p. 106)

^g^: except ‘emotional exhaustion’ (p. 107)

^h^: corrected for ‘distress’ (p. 169)

^i^: many dropouts (p. 170)

^j^: not corrected

^k^: corrected (Tables 2 and 3, p. 38–40)

^l^: + < 33% drop outs.

### The effects of MBSR on employees’ mental health

This section describes the effects of MBSR on employees’ mental health in the 23 studies reviewed. Several studies, e.g., Klatt et al. [78], Wolever et al. [82] and Geary and Rosenthal [86], also measured stress biomarkers (e.g., salivary cortisol, pulse rate and heart rate variability), but the effects of MBSR on such biomarkers fall outside the scope of our discussion. The mental health outcomes are presented in order of importance, taking into account two criteria. First and foremost is the level of evidence; the second criterion refers to the number of studies reporting a particular (significant or non-significant) result. When two effects have the same level of evidence from the same number of studies, they are described in alphabetical order.

Almost 35 mental/psychological outcomes were identified, some of them overlapping (e.g., stress and occupational stress, or mood and depression). Some outcomes were measured using different assessment instruments: e.g., stress level by the Perceived Stress Scale (PSS) and the Depression Anxiety Stress Scale (DASS); burnout by the Maslach Burnout Inventory (MBI) and the Professional Quality of Life (ProQOF) scale.

All results are presented in Table 3. Results classified as Levels 3, 4, or 5 and reported in a single study only are mentioned in Table 3, but not discussed further in the text.

**Table 3:** Outcomes/Results studies. 1) Duchemin et al. [76]; 2) Huang et al. [87]; 3) Amutio et al. [7]; 4) Taylor et al. [89]; 5) Roeser et al. [88]; 6 Wolever et al. [82]; 7) Pipe et al. [81]; 8) Klatt et al. [78]; 9) Cohen-Katz et al. Part II [73]; Cohen-Katz et al. Part III [74]; 10) Martín-Asuero et al. [90]; 11) Vega et al. [91]; 12) Frank et al. [85]; 13) Jennings et al. [83]; 14) Leroy et al. [9]; 15) Manotas et al. [80]; 16) Mackenzie et al. [79]; 17) Shapiro et al. [53]; 18) Klatt et al. [84]; 19) Beshai at al. [75]; 20) Geary and Rosenthal [86]; 21) Poulin et al. [8]; 22) Walach et al. [92]; 23) Horner et al. [77].

*Studies*	1	2	3	4	5	6	7	8	9	10	11	12	13	14	15	16	17	18	19	20	21	22	23	*Evidence level of outcome*	*Number of studies (outcome)*
Quality label of studies	HQ	HQ	MQ	MQ	MQ	MQ	MQ	MQ	MQ	MQ	MQ	MQ	MQ	MQ	MQ	MQ	MQ	LQ	LQ	LQ	LQ	LQ	LQ		
*Outcomes/results (numbered and described in the text)*																									
1 mindfulness	?		+		+	+		+	+	+	+	+	+	+	+				+				0	2	14
2 burnout^1^					+					+		0					0							2	9
* Emotional exhaustion*	0								+	+			0			+					+			2	
* Personal accomplishment*	0								+	0			+			+					0			2	
* depersonalization*	0								0	+			0			0								3	
3 stress level	+^a^	+				+		+							+		+		+	+			0	2	9
4 psychological distress		+					0		0			0			+		0			+		0		2	8
5 depression					+	0				0	+		0											2	5
6 (occupational) self-compassion					+							+					+		+					2	4
7 quality of sleep						+		0				+												2	3
8 relaxation			+													+					+			2	3
9 anxiety					+						+													2	2
10 occupational stress				+	+																			2	2
11 life satisfaction	0															+	0		+	+	+	0	0	3	8
12 mood										+			0											3	3
13 efficacy for regulating emotion at work				0								+												3	2
14 self-efficacy / locus of control													+									0		3	2
15 work engagement														+				0						3	2
*Outcomes/results (not numbered and not discussed in the text)*																									
angry reaction											+													3	1
attentional control											+													3	1
authentic functioning														+										3	1
daily physical symptoms													+											3	1
emotion regulation: cognitive reappraisal													+											3	1
empathy										+														3	1
focused attention and working memory capacity					+																			3	1
general hurry													+											3	1
job strain: job control and job demands		+																						3	1
subjective fatigue		+																						3	1
positive coping strategies																						+		4	1
spiritual experiences																				+				4	1
caring efficacy							0																	5	1
compassion towards others				0																				5	1
job satisfaction																0								5	1
negative coping strategies																						0		5	1
resilience (= emotional stability)																		0						5	1
ruminating and negative affects													0											5	1
sense of coherence																0								5	1
tendency to forgive				0																				5	1
work limitation						0																		5	1
work relationships									0															5	1

**0** no significance between-groups (TG and CG)

**+** significance between-groups (TG and CG)

**……….** Discussed in the text, chapter ‘the effects of MBSR on the mental health of employees’

Cohen-Katz et al. Part II [73] and Cohen-Katz et al. Part III [74]: one study, two articles

^1^: burnout and the three dimensions of burnout.

^a^: not significant on the Perceived Stress Scale (PSS); significant on the DASS (Depression Anxiety Stress Scale) stress subscale.

#### 1. Mindfulness

Fourteen of the studies reviewed measured the effect of MBSR on mindfulness [7, 9, 73, 75–78, 80, 82, 83, 85, 88, 90, 91] (see Table 3). In three studies [7, 9, 73], mindfulness significantly increased in the TG (within-group) and in the TG compared to the CG (between-groups). One study [77] reported no significant results; another [76] reported no results. The other nine studies mentioned a significant increase in the TG compared to the CG.

Nine studies [8, 53, 79, 81, 84, 86, 87, 89, 92] did not include a measure of mindfulness. In summary, it is plausible that MBSR significantly increases the amount of mindfulness (evidence Level 2).

#### 2. Burnout

Nine studies examined the effects of MBSR on burnout symptoms [8, 53, 73, 76, 79, 83, 85, 88, 90]. The three main symptoms of burnout are emotional exhaustion, (job-related) personal accomplishment, and depersonalization. Three of the studies [53, 85, 88] only reported on burnout in general. Five [8, 73, 76, 79, 83] reported the effects of MBSR on the three symptoms, but not on burnout in general. One study [90] dealt both with burnout in general and with the individual symptoms.

- Burnout in general: two studies reported a significant reduction in the TG [88, 90]; two reported no significant outcome [53, 85].
- Emotional exhaustion: two studies [8, 73] reported a significant reduction in the TG and in the TG compared to the CG. Two studies [79, 90] showed a significant reduction in the TG compared to the CG after the intervention. Two studies [76, 83] reported no significant outcome.
- (Job-related) personal accomplishment: levels increased significantly within-group and between-groups in three studies [73, 79, 83]. One study identified a significant increase in the TG [90], while two studies [8, 76] reported no significant differences.
- Depersonalization: one study [90] showed a significant reduction after the treatment in the TG and in the TG compared to the CG. In another study [79], depersonalization remained stable in the TG before and after the intervention (positive result), and increased significantly in the CG. Three other studies [73, 76, 83] reported no significant changes in depersonalization.

In summary, it is plausible that MBSR results in increased (job-related) personal accomplishment and decreased burnout in general and emotional exhaustion (evidence Level 2). There are indications that MBSR causes a decrease in depersonalization (cynicism and lack of empathy) (evidence Level 3).

#### 3. Stress level

Stress level, measured as perceived stress (mostly by the PSS), was investigated in nine studies [53, 75–78, 80, 82, 86, 87]. Eight studies reported a significant reduction in stress level after the intervention in the TG compared to the CG; one study [77] found no significant outcome. In summary, it is plausible that MBSR helps to reduce stress levels (evidence Level 2).

#### 4. Psychological distress

Eight studies investigated the effects of MBSR on psychological distress [53, 73, 80, 81, 85–87, 92]. Psychological distress was mostly measured by the Brief Symptom Inventory (BSI), which consists of 10 subscales reflecting different mood states (e.g., anxiety, depression, total mood disturbance). Three studies [80, 86, 87] showed a significant reduction in psychological distress in the TG and in the TG compared to CG; one study [81] mentioned a significant reduction in the TG. The other four studies reported no significant differences. In summary, it is plausible that MBSR results in a decrease of psychological distress (evidence Level 2).

#### 5. Depression

Depression was examined in five studies [82, 83, 88, 90, 91]. Two studies [88, 91] found a significant decrease of depression in the TG and in the TG compared to CG, one study [90] reported a significant reduction in the TG, and the other two studies [82, 83] mentioned no significant results. In summary, it is plausible that MBSR results in decreased levels of depression (evidence Level 2).

#### 6. (Occupational) self-compassion

Three studies addressed self-compassion, measured by the Self-Compassion Scale (SCS) [53, 75, 85], and one study addressed occupational self-compassion, measured by a modification of the SCS for teachers [88]. All four studies reported a significant increase in self-compassion in the TG and in the TG compared to the CG. Cohen-Katz et al.’s [74] qualitative study described an improvement in self-care resulting from MBSR: “I’m worrying about my own needs first, and trying to take care of them” (p. 82). Self-care can lead to feelings of guilt [74]: “they want to fix everyone else in the group!”, as one participant stated, “it’s the nurse in me!” (p. 85). In summary, it is plausible that MBSR leads to a significant increase in self-compassion (evidence Level 2).

#### 7. Quality of sleep

Three studies [78, 82, 85] investigated quality of sleep using the Pittsburgh Sleep Quality Index (PSQI), which consists of seven subscales: general (subjective) sleep quality, sleep latency, sleep duration, habitual sleep efficiency, sleep disturbances, use of medication to sleep, and daytime sleep-related dysfunction. Frank et al. [85] reported significant improvements in the TG compared to the CG in all aspects except for sleep efficiency and the use of medication to sleep. Wolever et al. [82] reported significant reductions in sleep difficulty in the TG compared to the CG.

Klatt et al. [78] found a significant improvement in the TG in terms of subjective sleep quality, sleep latency, sleep disturbances, and daytime dysfunction (i.e., four of the seven components of sleep quality). Changes in global sleep scores (PSQI) were significant for both the TG and the CG. Klatt et al. [78] suggested that the improvements in subjective sleep quality may be the study’s most important result.

In summary, it is plausible that MBSR gives rise to a significant increase in quality of sleep (evidence Level 2). However, it is not possible to provide insight into the different components of sleep quality.

#### 8. Relaxation

Three studies measured relaxation [7, 8, 79] and all found significant changes in relaxation in the TG compared to the CG. Cohen-Katz et al.’s [74] qualitative data also showed an increase in relaxation/calmness: “I’m feeling a greater calm and peace” (p. 82). Thus, it is plausible that MBSR produces a significant increase in relaxation (evidence Level 2).

#### 9. Anxiety

Two studies measured anxiety [88, 91] and both showed a significant decrease in anxiety in the TG compared to the CG. It is therefore plausible that MBSR causes a significant decrease in anxiety (evidence Level 2).

#### 10. Occupational stress

Two studies [88, 89] specifically measured occupational stress (i.e., stress employees experience on the job). Both reported a significant reduction in occupational stress after the intervention in the TG compared to the CG. In summary, it is plausible that MBSR causes a reduction in occupational stress level (evidence Level 2).

#### 11. Life satisfaction

Eight studies examined the effects of MBSR on general life satisfaction [8, 53, 75–77, 79, 86, 92]. Four [8, 75, 79, 86] reported significant positive changes in life satisfaction or general well-being in the TG compared to the CG. By contrast, four studies [53, 76, 77, 92] reported no significant improvement in terms of life satisfaction. In summary, there are indications that MBSR leads to an increase in life satisfaction (evidence Level 3).

#### 12. Mood

Mood was examined in two of the studies [83, 90]. Martín-Asuero et al. [90] used a short version of the Profile of Mood States (POMS), which consists of five subscales that measure negative emotions. They reported a significant reduction of total mood disturbance in the TG and in the TG compared to the CG. Jennings et al. [83] used the Positive and Negative Affect Schedule (PANAS), which measures positive and negative effects, and found no significant outcomes.

One qualitative study [74] described an improved ability to deal with difficult emotions following MBSR. In summary, there are indications that MBSR has a positive effect on mood (evidence Level 3).

#### 13. Efficacy in regulating emotions at work

Two studies [85, 89] measured employees’ perceived self-efficacy regarding their ability to regulate their emotions on the job. Frank et al. [85] reported a significant improvement in the TG compared to the CG; Taylor et al. [89] showed a significant increase in the TG. In summary, it is plausible that MBSR improves efficacy in regulating emotions at work (evidence Level 3).

#### 14. Self-efficacy/locus of control

Self-efficacy was investigated in two studies [83, 92]. Jennings et al. [83] mentioned a significant increase in the TG compared to the CG, while Walach et al. [92] reported no significant changes. In summary, there are indications that MBSR causes an increase in self-efficacy in general (evidence Level 3).

#### 15. Work engagement

Work engagement was measured in two studies [9, 84]. Leroy et al. [9] showed a significant increase in work engagement for the group as a whole between T1 and T2; the increase in the TG compared to the CG appeared to be significantly higher. Klatt et al. [84] reported a significant increase in work engagement in the TG, mostly induced by the vigor subscale (a subscale of the Utrecht Work Engagement Scale, UWES). In summary, there are indications that MBSR produces a significant increase in work engagement (evidence Level 3).

## Discussion

To the best of our knowledge, this is the first review of the mental health of employees across different occupational sectors. We identified 24 articles representing 23 studies on the effects of MBSR (2 high quality, 15 medium quality, and 6 low quality) published before October 2015.

Demanding workplace challenges can produce stress and symptoms of burnout [93]. In the Netherlands, the professionals reporting the highest levels of work pressure and stress are teachers and healthcare providers [94], and teachers appear to have the highest burnout percentage in the Dutch workforce [95]. Burnout is a major cause of loss of engagement, disease, and disability [96].

There is a great need for useful, practical workplace interventions that could reduce stress and enhance work engagement. Kabat-Zinn [18], who introduced the concept of mindfulness, suggested its possible use as a person-centered intervention. This study was aimed at investigating empirically whether MBSR and MBCT indeed contribute to employees’ mental health.

### General findings

#### Measuring mindfulness

Fourteen of the 23 studies reviewed [7, 9, 73, 75–78, 80, 82, 83, 85, 88, 90, 91] measured levels of mindfulness; 9 did not. This is striking because, as Cohen-Katz et al. [73] points out, measuring mindfulness is important to determining whether an MBSR program has successfully taught what it was designed to teach. Mindfulness skills may be the mediating factor for individual outcomes such as stress reduction, relaxation, and empathy [97]. Indeed, Baer [29] highlighted several mechanisms that might account for how mindfulness skills can reduce symptoms and bring about behavioral change: exposure, cognitive change, self-management, relaxation, and acceptance.

Three different mindfulness measures were used in the studies we reviewed: five used the Mindfulness Attention Awareness Scale (MAAS), eight used the Five Facets Mindfulness Questionnaire (FFMQ) with five subscales, and one used the Cognitive and Affective Mindfulness Scale-Revised (CAMS-R). These three scales correspond with different definitions of mindfulness. In the MAAS, mindfulness is operationalized as a one-dimensional construct involving attention and awareness. The FFMQ, which encompasses five dimensions of mindfulness, also includes an attitudinal component. The CAMS-R, which assesses four elements of mindfulness, yields only one score [98]. The use of different ways of operationalizing mindfulness makes it difficult to compare its effects. In addition, mindfulness can be said to be both an outcome variable of interest and an important mechanism of the therapeutic agent.

The advantage of the FFMQ is the fact that Baer’s [29] five subscales result in a sophisticated measurement of mindfulness. As it is based on a factor analysis of items from the five most widely used mindfulness questionnaires, it can also be considered particularly robust [99, 100].

#### Other outcomes

Most of the variables were assessed using negative symptom-focused outcome measures (e.g., burnout, stress level, psychological distress, depression, anxiety), while some used positive symptom-focused outcome measures (e.g., quality of sleep, relaxation, work engagement, job satisfaction). There were hardly any symptom-focused outcome measures relating to work performance (e.g., caring efficacy, work behavior, work performance, workability).

Few variables, except for mindfulness skills (observing, describing, acting with awareness, non-judging, non-reactivity), were assessed using process-focused measures. They may have been more suitable for capturing the mechanisms by which mindfulness practice leads to specific outcomes (e.g., (occupational) self-compassion, (occupational) self-efficacy, sense of coherence, coping strategies, ruminating). Specifically, four of the studies [53, 75, 85, 88] reported a significant increase in self-compassion in the TG compared with the CG. In another study, Shapiro et al. [11] reported that MBSR weakens stimulus-response relationships, thereby reducing reflexive behavior. This was associated with increased levels of self-management, self-efficacy, and self-care.

Self-care can be challenging for healthcare professionals and others engaged in people-centered work (e.g., teachers), as their job is to care for others. A lack of self-care can eventually undermine health and, as a result, sustainable employability [101]. Thus, employees’ self-care is a highly important topic that deserves more attention within organizations. Our findings suggest that it might be increased by means of mindfulness training.

Behavioral variables, such as assessments of the quality and quantity of formal and informal meditation (e.g., frequency and intensity of practice) were measured in only three studies [9, 74, 92].

Self-reported measures are inherently biased because participants who have invested a lot of time and energy in a treatment program, such as MBSR, are less likely to give negative evaluations [102]. Therefore, behavioral reports by relevant others may be a useful addition.

A few outcomes in our review refer to perceptions of work characteristics (e.g., work relationships, job control, job demands). However, many other possible work-related perceptions were not measured (e.g., work pressure, emotional load, feedback, autonomy, learning opportunities). Further empirical work on the effects of mindfulness on perceptions of work could be revealing.

Future research on mindfulness treatments for employees may benefit from assessing a diversity of outcomes: negative and positive symptom-focused measures of mental health (mood, recovery need, job satisfaction, work engagement) and work performance (positive and negative work behavior, absence from work); process-focused outcome measures; behavioral measures of (in)formal practices; behavioral reports by relevant others (e.g., colleagues); and outcomes on work-related perceptions. Finally, future research may include outcomes differentiating between elements of mindfulness interventions, as these remain something of a black box thus far in terms of their working mechanisms.

#### MBCT

Our search method, which included both MBSR and MBCT programs, uncovered only one study [75] exploring the effects of a modified MBSR program in combination with some aspects of MBCT on employees. MBCT is often used in clinical settings to prevent depression relapse [20]. However, it could also be useful for employees, especially those who experience non-productive or irrational thoughts, which can cause stress. Two examples of excluded pre-post studies focusing exclusively on the effects of MBCT on employees are Ruths et al. [24] and Schenström et al. [103]. Future research on the impact of MBCT on employees is needed to compare its efficacy to that of MBSR.

#### Contraindications

None of the studies we reviewed reported that MBSR training negatively affected employees. The absence of empirical evidence on potentially harmful effects of MBSR [104] does not mean it is good for everyone in every situation; instead, potentially harmful effects should be thoroughly evaluated in future work. Ruths et al. [24], who investigated the effects of MBCT on 27 mental-health professionals in a pre-post study (which was therefore excluded from our study), mentioned depersonalization and initial mood deterioration as potentially harmful effects of meditation. Participants with severe trauma or those at risk for psychosis might be at elevated risk [49]. Dobkin et al. [104] made several recommendations in this regard, including pre-program screening to “assess the patient’s ability to: (1) contain affect; (2) listen and respond in the present; (3) utilize instructional audio tapes and follow classroom instruction; (4) remain in the classroom; (5) practice yoga or equivalent; and (6) organize thoughts, manage logistics, and time commitment” (p. 4). Nonetheless, there is little empirical research on and evidence for the harmful effects of mindfulness training [49].

### Methodological issues

Despite the promising findings of the studies in our review, our approach has some limitations which make it difficult to draw strong conclusions about the effects of MBSR on employees.

#### Meta-analysis versus systematic review

We chose to conduct a systematic review instead of a meta-analysis for four reasons. First, the quality of the studies conducted so far is limited; we only found 2 studies that could be classified as of high methodological quality, while 15 were of medium quality and 6 were of low quality (Table 2).

Second, mindfulness training is an emergent, relatively uncharted field of investigation, with a broad variety of outcome measures. Only 7 variables were examined in four or more studies, 8 in two or three studies, and 22 in one study only (Table 3). We intended to explore several negative and positive symptom-focused outcomes on mental health and work performance; in particular, positive and negative process-focused outcomes and positive and negative outcomes related to work characteristics. Furthermore, we wanted to use quantitative and qualitative data.

Third, the samples in 11 of the studies reviewed were relatively small, ranging from just 10 to 24 participants.

Finally, the 2 high-quality studies and 15 medium-quality studies appeared to have similar outcome variables in a few cases (as shown in Table 1).

#### The (lack of) clarity around the outcome variables

We identified many mental/psychological outcomes which sometimes partly overlapped (e.g., stress and occupational stress; mood and depression; efficacy in regulating emotions at work and self-efficacy/locus of control). Occupational stress refers to only one cause of stress, while depression is just one aspect of mood, measured by five subscales (tension-anxiety, depression-dejection, anger-hostility, vigor-activity, and fatigue-inertia) of the Profile of Mood States (POMS). In a similar vein, efficacy in regulating emotions at work appears to be a single dimension of self-efficacy/locus of control.

In some cases, outcomes were measured using different assessment instruments. For example, mood was measured by the POMS, which consists of five subscales, and by the Positive and Negative Affect Scale (PANAS), which consists of two corresponding subscales. Mindfulness was measured by the one-dimensional MAAS, the five-dimensional FFMQ or the CAMS-R, with its four elements of mindfulness.

Sometimes the distinction between outcomes was unclear. For example, there is no clear distinction between mood, measured by a short version of the POMS [90], and psychological distress, measured by the Brief Symptom Inventory (BSI). The BSI consists of 10 subscales reflecting different mood states, such as anxiety, depression, and total mood disturbance. The POMS consists of five subscales measuring negative emotions.

As already mentioned, three of the studies reviewed [53, 85, 88] reported only on burnout in general. Five studies [8, 73, 76, 79, 83] reported on the three dimensions of burnout but not on burnout in general. One study [90] dealt with both types of outcomes.

There is a need for unambiguous, clear outcomes that are logically clustered (e.g., process and effect outcomes; mental-health and work-performance outcomes; positive and negative outcomes; work-related perceptions outcomes; individual and organizational outcomes) and measured by reliable and valid assessment instruments.

#### Publication bias

All the studies except one [77] reported statistically significant outcomes (Table 3). Three studies (12.9%) [76, 81, 92] mentioned only one significant outcome, three (12.9%) [7, 53, 84] mentioned two significant outcomes, and the other 16 studies (68.8%) reported at least three significant outcomes. The multitude of significant results ought to be seen in the context of the small sample sizes used in most of the studies, and the medium and low methodological quality of 15 and 6 studies, respectively, leaving them with relatively low statistical power. It may also reflect some form of publication bias, as studies with positive results tend to be published more easily than studies with negative results [105].

#### Short- and long-term effects

Little research has considered the long-term effects of mindfulness interventions. Fourteen of the studies reviewed measured short-term effects only [8, 53, 75–80, 82–85, 90, 91], as their final measurements were conducted immediately after the intervention. The other nine studies measured the effects over a longer period: eight weeks to four months post-intervention for six studies [9, 73, 87–89, 92], and almost one year post-intervention in three studies [7, 81, 86]. Mindfulness training for employees should ideally result in sustainable long-term effects. Therefore, both short- and long-term effects of the MBSR program for employees need to be considered in future research.

#### Length of treatment program

Nine of the studies reviewed [8, 75–82] used shortened MBSR treatment programs. Two used a modified MBSR program [83, 84], and one used an extended MBSR program [7]. The other 11 studies used the usual MBSR program [9, 53, 73, 85–92].

Due to the different lengths of the programs, we cannot safely conclude anything about the effects of program length on the results. However, it is important to note that seven of the eight shortened MBSR studies (all except Pipe et al. [81]) only measured short-term effects. As Carmody and Baer [106] stated, “the effect of variation in class hours on outcomes has not been systematically studied” (p. 627). They did not find a significant correlation between the number of in-class hours and the mean effect size in clinical or non-clinical samples. Studies on very brief mindfulness interventions in lab settings found temporary effects on emotion [107], mood, and stress [108, 109]. Increases tend to be found in state (present) mindfulness rather than trait (more permanent) mindfulness [5]. Bear, Carmody, and Hunsinger [110] revealed that structural changes in perceived stress did not occur until week four of the mindfulness training. Changing problematic, automatic patterns of thought and behavior takes time, and mindfulness is no quick solution.

Carmody and Bear suggested that “adaptations that include less class time may be worthwhile for populations for whom reduction of psychological distress is an important goal and for whom longer time commitment may be a barrier to their ability or willingness to participate” [106]. In Shapiro et al.’s [53] study, 8 of the 18 participants dropped out of the treatment group. The authors concluded that “adding a 2-hour intervention plus daily home practice to an already demanding schedule may not be feasible for a substantial number of health care professionals” (p. 172). Kabat-Zinn [111] emphasized the importance of flexibility in the MBSR program in different contexts. The original MBSR program with eight 2.5-hour sessions, a 7-hour day of silence, and 45 minutes of daily practice at home seems to be too demanding for busy workers [17]. Chaskalson et al. [17] indicated that although the number of organizations offering MBSR is increasing, they all use a format with fewer and shorter sessions. There is a growing need to adapt the duration and dose of the MBSR program to different workplace contexts, in order to better get to grips with its effectiveness.

Another consideration regarding the treatment schedule is whether parts of the program are difficult to adhere to (e.g., regular class attendance, daily home practice, formal and informal meditation exercises). Only Cohen-Katz et al. [74] collected qualitative data to this end. More research is needed on this issue, and to determine whether a shortened version of the MBSR program would produce the same short- and long-term effects.

#### Level of evidence

The level of evidence that we were able to obtain was based on the number of studies investigating a certain variable (see Table 3) and the quality of those studies (see Table 2). Only seven variables were examined in four or more studies, eight variables in two or three studies, and 22 variables in just one study (Table 3). We only found evidence that met the requirements for Levels 2 through 5; we found no evidence meeting the requirements for Level 1. Future studies should seek to generate Level 1 evidence from high-quality RCTs.

#### Small sample sizes

The sample sizes of 11 studies [7, 8, 53, 73, 76–79, 81, 85, 92] were relatively small: the group sizes (TG and CG) ranged from 10 to 24 non-clinical participants. As statistical significance is essential to the interpretation of findings, future empirical work should be conducted using larger sample sizes.

#### Homogeneous and heterogeneous samples

The research populations included in our review mainly comprised healthcare professionals (12 studies) [7, 53, 73, 76, 77, 79–81, 84, 86, 90, 91]. Five studies examined teachers [75, 83, 85, 88, 89].

Five studies [7, 8, 79, 81, 85] had homogeneous participant samples in terms of occupation. Three studies [83, 88, 89] had broadly homogeneous samples, in that they investigated related occupations (i.e., elementary, secondary, and high school teachers). The other studies [9, 53, 73, 75–78, 80, 82, 84, 86, 87, 90–92] had mixed/heterogeneous samples with various participant occupations.

Future research may benefit from using more homogeneous samples (e.g., workers in healthcare, education, or finance), as these groups have specific demands and challenges with respect to the outcomes of MBSR treatment.

#### Employees

As previously mentioned, the samples in the studies reviewed largely consisted of healthcare professionals and elementary, secondary, and high school teachers. They do not represent the target population of employees in general. Therefore, more research across different occupational sectors is needed to generalize our conclusions. It may be that including terms such as ‘business’ or ‘corporate’ in our search string would have yielded additional references.

#### Self-selection bias

A major limitation of all the studies reviewed is self-selection, as voluntary participation by employees may result in somewhat biased samples. Specifically, the characteristics of employees who participate in such research (e.g., motivation, sensitivity to the MBSR program, discipline) may differ from those who do not. Self-selection bias thus complicates the evaluation of the MBSR program and the interpretation of results.

#### Quantitative and qualitative data

Future research may benefit from mixed-methods approaches that combine quantitative and quantitative data. To date such a combination is lacking in the literature.

Twenty of the studies reviewed contained mostly quantitative data, while only three [73, 89, 92] contained a substantial amount of qualitative data. We found one qualitative review, by Morgan et al. [57], which synthesized 14 qualitative studies on the experiences of health-care professionals and students with mindfulness training and discussed issues such as self-compassion, initial challenges to practice, training focus, and participant motivation.

Since mindfulness research on employees across occupational sectors is a relatively new phenomenon, qualitative data are needed to be able to investigate in depth whether an intervention significantly affects process-focused personal measures (e.g., mindfulness skills, coping strategies) and to capture the mechanisms by which mindfulness practice leads to specific outcomes. For example, unresolved or suppressed emotions may resurface during mindfulness training, contributing to self-consciousness. As Cohen-Katz et al. [74] write, “For the participants (i.e. nurses), becoming aware of the wound was both painful and ultimately extremely useful, helping them to move forward in their lives” (p. 86).

Qualitative data are also needed to thoroughly investigate key aspects of the mindfulness training program and to examine those factors that lead to successful implementation in an organization (e.g., allocating staff time to participate, support of superiors).

#### Additional organizational intervention

Stress is the result of a complex interaction between environmental factors (work and personal circumstances) and the individual [96, 112]. Interventions designed to reduce stress have generally targeted either the relevant environmental factors or the individual, personal factors.

Person-centered interventions seem to be only partly effective in influencing mental health and well-being [113]. An integrated approach is needed that considers not only the person, but the work context as well [114]: for instance, by combining a person-centered intervention such as mindfulness with an additional organizational intervention. None of the studies reviewed used such an additional intervention, which may enhance the effects of the mindfulness intervention.

#### MBSR program and instructor skill

Specific characteristics of the programs used, such as the instructors’ skill levels, may influence the efficacy of MBSR treatment. None of the 23 studies described the MBSR program in terms of its form, content, procedures, or material. Several studies described the instructors as “experienced,” but the term was not well-defined. Future studies would benefit from a full explanation of both the MBSR program and the instructors’ skill levels.

## Conclusions

Our systematic literature search of PsycINFO, PubMed and CINAHL resulted in 24 articles representing 23 studies on the effects of MBSR and MBCT. Their methodological quality varied: 2 were of high quality, 15 of medium quality and 6 of low quality. Given the low number of studies and relatively low methodological quality, it is clear that research on the effects of mindfulness on employees remains a relatively uncharted area.

The outcomes of the studies reviewed suggest that MBSR may help to improve employees’ psychological functioning. However, no firm conclusions can be drawn about the effects of specific mindfulness programs for different groups and/or under specific conditions.

The strongest outcomes were decreased levels of emotional exhaustion (a dimension of burnout), stress, psychological distress, depression, anxiety, and occupational stress.

We also found a significant increase in mindfulness, personal accomplishment (a dimension of burnout), (occupational) self-compassion, quality of sleep, and relaxation.

## Supporting information

S1 PRISMA Checklist PRISMA checklist items reported and their location within the text. (PDF)
